# Type 2 Diabetes and Colorectal Cancer Risk

**DOI:** 10.1001/jamanetworkopen.2023.43333

**Published:** 2023-11-14

**Authors:** Thomas Lawler, Zoe L. Walts, Mark Steinwandel, Loren Lipworth, Harvey J. Murff, Wei Zheng, Shaneda Warren Andersen

**Affiliations:** 1 University of Wisconsin Carbone Cancer Center, Madison; 2 Department of Population Health Sciences, School of Medicine and Public Health, University of Wisconsin-Madison; 3 International Epidemiology Field Station, Vanderbilt Institute for Clinical and Translational Research, Rockville, Maryland; 4 Division of Epidemiology, Department of Medicine, Vanderbilt Epidemiology Center, Vanderbilt-Ingram Cancer Center, Vanderbilt University School of Medicine, Nashville, Tennessee; 5 Department of Medicine, Vanderbilt University School of Medicine, Nashville, Tennessee

## Abstract

**Importance:**

Type 2 diabetes and colorectal cancer (CRC) disproportionately burden indviduals of low socioeconomic status and African American race. Although diabetes is an emerging CRC risk factor, associations between diabetes and CRC in these populations are understudied.

**Objective:**

To determine if diabetes is associated with CRC risk in a cohort representing understudied populations.

**Design, Setting, and Participants:**

This cohort study uses data from the prospective Southern Community Cohort Study in the US, which recruited from 2002 to 2009 and completed 3 follow-up surveys by 2018. Of about 85 000 participants, 86% enrolled at community health centers, while 14% were enrolled via mail or telephone from the same 12 recruitment states. Participants with less than 2 years of follow-up, previous cancer diagnosis (excluding nonmelanoma skin cancer) at enrollment, missing enrollment diabetes status, diabetes diagnosis before age 30, and without diabetes at enrollment with no follow-up participation were excluded. Data were analyzed from January to September 2023.

**Exposures:**

Physician-diagnosed diabetes and age at diabetes diagnosis were self-reported via survey at enrollment and 3 follow-ups.

**Main Outcomes and Measures:**

Diabetes diagnosis was hypothesized to be positively associated with CRC risk before analysis. Incident CRC was assessed via state cancer registry and National Death Index linkage. Hazard ratios and 95% CIs were obtained via Cox proportional hazard models, using time-varying diabetes exposure.

**Results:**

Among 54 597 participants, the median (IQR) enrollment age was 51 (46-58) years, 34 786 (64%) were female, 36 170 (66%) were African American, and 28 792 (53%) had income less than $15 000 per year. In total, 289 of 25 992 participants with diabetes developed CRC, vs 197 of 28 605 participants without diabetes. Diabetes was associated with increased CRC risk (hazard ratio [HR], 1.47; 95% CI, 1.21-1.79). Greater associations were observed among participants without colonoscopy screening (HR, 2.07; 95% CI, 1.16-3.67) and with smoking history (HR, 1.62; 95% CI, 1.14-2.31), potentially due to cancer screening differences. Greater associations were also observed for participants with recent diabetes diagnoses (diabetes duration <5 years compared with 5-10 years; HR, 2.55; 95% CI, 1.77-3.67), possibly due to recent screening.

**Conclusions and Relevance:**

In this study where the majority of participants were African American with low socioeconomic status, diabetes was associated with elevated CRC risk, suggesting that diabetes prevention and control may reduce CRC disparities. The association was attenuated for those who completed colonoscopies, highlighting how adverse effects of diabetes-related metabolic dysregulation may be disrupted by preventative screening.

## Key Points

**Question:**

What is the association of diabetes with colorectal cancer (CRC) risk in an African American, low-income population?

**Findings:**

In this cohort study of 54 597 adults, a diabetes diagnosis was associated with a 47% increased risk of developing CRC compared with participants without a diabetes diagnosis. This association was greater for participants without recent colonoscopy screenings and participants with a more recent diabetes diagnosis.

**Meaning:**

These findings suggest that given the emerging association between diabetes and elevated risk for colorectal cancer, screening via colonoscopy for individuals with diabetes may help to mitigate risk.

## Teaser

This cohort study evaluates the association of diabetes with colorectal cancer risk in an African American, low-income population.

## Introduction

Type 2 diabetes is a condition of progressive insulin dysregulation, typically occurring alongside metabolic dysfunctions including hyperglycemia and hyperinsulinemia.^1^ In the US, an estimated 37.3 million people have diabetes, as indicated by abnormal fasting plasma glucose or hemoglobin A_1c _(HbA_1c_).^2^ Black adults have a disproportionate diabetes burden, with an age-adjusted prevalence of 16.8% (95% CI, 15.3-18.5) compared with 11.2% (95% CI, 9.5-13.2) in White adults.^2^ Both prevalence^3,4^ and incidence^5^ of diabetes are rising more in Black than White adults, suggesting widening racial disparities. Socioeconomic disparities by both education and household income are also evident.^2,4^

Epidemiological and biological evidence suggests that individuals with diabetes diagnoses may be at increased risk for colorectal cancer (CRC).^6,7^ The metabolic dysregulation that occurs in diabetes may contribute to CRC carcinogenesis and proliferation through inflammation and oxidative stress-driven signaling alterations.^6,8^ Meta-analyses report associations between type 2 diabetes and increased risk of CRC, with risk ratios ranging from 1.2 to 1.4.^9,10,11,12^ CRC shares many risk factors with diabetes, including low socioeconomic status (SES), Black race, obesity, and smoking.^2,13,14,15^ However, effect modification of the associations between diabetes and CRC risk by shared risk factors, including racial identity and SES, have not yet been fully characterized in biomedical studies.

The Southern Community Cohort Study (SCCS) is a prospective cohort study designed to investigate cancer-related health disparities by oversampling low-income and African American participants. Although these population groups tend to experience disproportionate disease burden of both diabetes and CRC, they are underrepresented in prospective epidemiologic studies, particularly in the literature exploring the connection between diabetes and CRC risk. The present study aims to assess associations between diabetes and CRC and evaluate whether there is effect modification by risk factors, CRC screening participation, or duration of diabetes burden.

## Methods

### Study Sample

Data were obtained from the SCCS, a prospective cohort study including participants from 12 states in the southeastern US. The design and rationale for the SCCS has been described previously.^16^ Approximately 85 000 English-speaking participants aged 40 to 79 years were enrolled between 2002 and 2009, with 86% of participants enrolled through community health centers and the remaining 14% enrolled via mail or telephone. Upon enrollment, participants completed questionnaires concerning demographics, medical history, family history of disease, diet, and physical activity. Racial identity was obtained via participant self-report. Body mass index (BMI) was calculated from height and weight measurements that were abstracted from medical records, if taken at enrollment, or were self-reported by participants on the enrollment questionnaire.^16^ Follow-up was completed via questionnaires mailed to participants at approximately 5-year intervals, with the third follow-up completed in 2018 (eFigure in Supplement 1). Incident CRC diagnosis after enrollment was identified through linkage to state cancer registries and the National Death Index. Linkage dates varied by state between the years 2016 to 2020, thus the censoring dates for participants vary by location. CRC was defined using the *International Classification of Diseases–Oncology, *third edition, codes C180 through C189, C199, and C209. All study activities conformed to the tenets of the Declaration of Helsinki, and all participants provided written informed consent. Institutional review board approval was provided by Meharry Medical College and Vanderbilt University Medical Center. The study sample and all subsequent analyses were prepared in accordance with Strengthening the Reporting of Observational Studies in Epidemiology (STROBE) reporting guidelines for cohort studies.

### Participant Eligibility and Data Characteristics

Participants were excluded who had less than 2 years of follow-up after enrollment (1676 participants), self-reported a previous cancer diagnosis at SCCS enrollment other than nonmelanoma skin cancer (6631 participants), were missing diabetes status at enrollment (465 participants), or received a diabetes diagnosis before age 30 years (1250 participants) to remove participants with type 1 diabetes. Moreover, to limit the effects of misclassification of the study exposure, we excluded 18 880 participants who reported not having diabetes at enrollment and did not participate in any follow-up interviews. Among the full analytic cohort (73 477 participants), 50 315 participants (68.4%) completed at least 1 follow-up survey, and 28 774 (39.2%) participated in follow-up 3.

### Diabetes Assessment

Self-reported physician’s diagnosis of diabetes was assessed upon enrollment and in all follow-up questionnaires. Participants who self-reported diabetes were subsequently asked to report their age at first diagnosis. At follow-up, participants were asked to report whether they had completed a blood test to check for diabetes since study enrollment. SCCS validation studies demonstrated high specificity for self-reported diabetes, with 96%^17^ and 98%^18^ of self-reported diagnoses confirmed via medical records and/or HbA_1c_ testing. Sensitivity is lower in the SCCS, around 42%,^18^ consistent with high rates of undiagnosed diabetes in the US.

### Statistical Analysis

Participant characteristics were tabulated by diabetes and incident CRC status. Associations between diabetes status and incident CRC risk were assessed using Cox proportional hazards models with age as the time scale to calculate hazard ratios (HR) and 95% CIs for overall CRC incidence and separately by tumor location. Diabetes status was modeled using a time-varying exposure variable. Separate estimates were obtained for any diabetes diagnosis, prevalent diabetes at enrollment, and incident diabetes during study follow-up. Entry time in Cox models was defined as age at enrollment and exit time as age at CRC diagnosis, age at death, or the end of follow-up in the linked state cancer registry, whichever came first. Participants who did not report diabetes at baseline were censored at their age at the last follow-up in which they participated. All models were adjusted for the following potential confounders that were assessed at enrollment: self-reported race and ethnicity (African American, White, or other, which includes American Indian or Alaska Native, Asian or Pacific Islander, Hispanic/Latino, and more than 1 race and ethnicity), sex (male or female), enrollment source (community health center or mail/telephone), colorectal cancer screening at enrollment (ever or never completed colonoscopy or sigmoidoscopy), insurance coverage (yes or no), yearly household income (<$15 000, $15 000-49 999, or ≥$50 000), education (<high school, high school, or >high school), smoking history (never, former, or current), heavy alcohol consumption (≥1 drink/day for women or ≥2 drinks/day for men), BMI (<25, ≥25 to <30, ≥30 to <35, ≥35 to <40, or ≥40.0; BMI is calculated as weight in kilograms divided by height in meters squared), and family history of CRC in a first-degree relative (yes or no). To determine whether adjusting for adiposity changed the association between diabetes and CRC risk, models were constructed with and without BMI as a covariate. Because missing data for covariates were relatively infrequent (approximately 1%-2% of observations), missing values were imputed using the race-specific and sex-specific mode (categorical variables) or median (continuous variables). For participants who self-reported diabetes diagnosis during follow-up but did not provide the age at first diagnosis, diagnosis was assumed to have occurred at the midpoint between questionnaires. The proportional hazards assumption was assessed for all covariates using log-log survival curves and was considered met. Statistical analyses were completed January to September 2023, using SAS version 9.4 (SAS Inc). A 2-sided *P* value of less than .05 was used as the threshold for statistical significance.

The association between diabetes and CRC risk was investigated in subgroups defined by sex, income, race and ethnicity, obesity, and smoking history. Tests for statistical interaction were completed by adding an interaction term to the fully adjusted model and evaluating the *P* value for significance. Most of our analyses exclude participants who did not report diabetes diagnosis at cohort enrollment and did not have any follow-up questionnaire data. To evaluate how the exclusion changed our results, we conducted a sensitivity analysis among the full analytic cohort where we included participants who did not report diabetes at cohort enrollment and did not have follow-up questionnaire data.

To investigate how CRC screening via colonoscopy changes the association between diabetic metabolic dysregulation and CRC risk, we performed a sensitivity analysis limited to participants who reported never undergoing colonoscopy at follow-up 2 and/or 3. We also performed an analysis among participants who reported ever completing colonoscopy at follow-up 2 and/or 3 and reported ever undergoing a blood glucose test to screen for diabetes. We focused on CRC screening at follow-up 2 and 3 because the study questions were more comprehensive and included detailed categorization according to screening frequency.

To identify a critical window of exposure in the association between diabetes and CRC risk, we evaluated the association between diabetes duration and CRC among participants with diabetes diagnoses. Total diabetes duration was calculated beginning from the individual’s age at diabetes diagnosis and terminating at the age of cancer diagnosis, death, or end of follow-up. For this analysis, participants were excluded who had missing age at diabetes diagnosis (340 participants) or less than 2 years of follow-up after diabetes diagnosis (181 participants).

## Results

### Baseline Characteristics of the Sample

In total, 54 597 participants were analyzed (median [IQR] enrollment age, 51 [46-58] years), and most were female (34 786 participants [64%]), African American (36 170 participants [66%]), and had an annual household income less than $15 000 (28 792 participants [53%]). At enrollment, 14 998 (28%) reported prevalent diabetes, and 10 994 (20%) reported incident diabetes during follow-up. Participants with prevalent diabetes were older (median [IQR] age, 54 [48-61] years) than participants with incident diabetes (50 [45-56] years) or with no self-reported diabetes (50 [45-57] years).

Participants with diabetes were more likely to be female and African American, and have obesity, lower incomes, and lower educational attainment than participants who did not report diabetes during study follow-up (Table 1). Compared with participants without diabetes, those with prevalent diabetes at enrollment were less likely to be a current smoker or heavy drinker, and more likely to have health insurance and participate in CRC screening. Characteristics of persons with diabetes relative to persons without diabetes remained similar in the full analytic cohort (73 477 participants) (eTable 2 in Supplement 1). There were 486 participants with incident CRC diagnoses during the study period. Participants with CRC were older at enrollment and more likely to report African American racial identity, lower educational attainment, and lower participation in CRC screening than those without CRC (eTable 1 in Supplement 1).

**Table 1.** Characteristics of Southern Community Cohort Study Participants Stratified by Timing of Diabetes Diagnosis

Characteristic	Participants, No. (%)		
	No diabetes (n = 28 605)	Prevalent diabetes (n = 14 998)	Incident diabetes (n = 10 994)
Enrollment age, median (IQR), y	50 (45-57)	54 (48-61)	50 (45-56)
Sex			
Female	17 867 (62)	9656 (64)	7263 (66)
Male	10 738 (38)	5342 (36)	3731 (34)
Enrollment source			
Community health center	22 909 (80)	13 247 (88)	9275 (84)
General population	5696 (20)	1751 (12)	1719 (16)
Race and ethnicity			
African American	17 257 (60)	10 578 (71)	8335 (76)
White	10 141 (35)	3818 (25)	2318 (21)
Other^a^	1207 (4)	602 (4)	341 (3)
Educational attainment			
<High school	6228 (22)	5059 (34)	3066 (28)
High school	9116 (32)	4827 (32)	3721 (34)
>High school	13 261 (46)	5112 (34)	4207 (38)
Household income, $			
<15 000	13 708 (48)	9071 (60)	6013 (55)
15 000-49 999	10 662 (37)	4977 (33)	4096 (37)
≥50 000	4235 (15)	950 (6)	885 (8)
Insurance, yes	18 019 (63)	10 084 (67)	6644 (60)
Colorectal cancer screening, yes	9135 (32)	5731 (38)	3363 (31)
Family history of colorectal cancer	1960 (7)	1063 (7)	689 (6)
Body mass index ≥30^b^	10 617 (37)	9877 (66)	6122 (56)
Smoking status			
Never	11 169 (39)	6234 (42)	4227 (38)
Former	6551 (23)	4411 (29)	2495 (23)
Current	10 885 (38)	4353 (29)	4272 (39)
No and moderate alcohol consumers^c^	23 545 (82)	13 732 (92)	9166 (83)
Physical activity, median (IQR), MET-hrs/d	19 (11-31)	15 (8-25)	18 (10-31)
Sedentary time, median (IQR), h	9 (6-12)	9 (6-12)	9 (6-12)
Age at CRC diagnosis, median (IQR), y (n = 486)	61 (56-67)	65 (58-71)	61 (54-70)
CRC site (n = 486)			
Colon	150 (76)	151 (76)	71 (79)
Rectum	47 (24)	48 (24)	19 (21)
Tumor stage (n = 371)			
0	5 (3)	7 (4)	2 (3)
I	48 (32)	44 (28)	19 (30)
II	38 (26)	36 (23)	14 (22)
III	38 (26)	39 (25)	15 (23)
IV	19 (13)	33 (21)	14 (22)

Abbreviations: CRC, colorectal cancer; MET, metabolic equivalent.

^a^ Includes American Indian or Alaska Native, Asian or Pacific Islander, Hispanic/Latino, participants of more than 1 race and ethnicity, and participants from other racial or ethnic groups.

^b^ Body mass index is calculated as weight in kilograms divided by height in meters squared.

^c^ No to moderate alcohol consumption defined as alcohol intake of 1 drink/d or less for women or 2 drinks/d or less for men.

### Diabetes Status and CRC Risk

In total, 289 of 25 992 participants with diabetes developed CRC, vs 197 of 28 605 participants without diabetes. Diabetes was associated with increased risk of CRC (HR, 1.47; 95% CI, 1.21-1.79) (Table 2). Associations were similar with and without adjustment for BMI. The associations between diabetes and CRC risk were similar in analyses stratified by colon and rectal cancer sites but were somewhat greater among participants with prevalent diabetes diagnosis at enrollment (HR, 1.59; 95% CI, 1.28-1.97) compared with participants with incident diabetes (HR, 1.28; 95% CI, 0.99-1.66). Associations were greater for females (HR, 1.59; 95% CI, 1.24-2.04) compared with males (HR, 1.27; 95% CI, 0.93-1.75). In a sensitivity analysis which included all participants in the full analytic cohort, the association between diabetes and CRC was attenuated toward the null (HR, 1.14; 95% CI, 0.98-1.33) (eTable 3 in Supplement 1).

**Table 2.** Associations Between Diabetes and Colorectal Cancer Incidence Stratified by Anatomic Site^a^

Diagnosis	Colorectal cancer incidence			Colon cancer incidence			Rectal cancer incidence		
	Person-years	Cases^b^	HR (95% CI)^c^	Person-years	Cases^b^	HR (95% CI)^c^	Person-years	Cases^b^	HR (95% CI)^c^
All participants (n = 54 597)									
No diabetes	331 035.8	197	1 [Reference]	330 636.5	150	1 [Reference]	329 690.0	47	1 [Reference]
Diabetes at enrollment or follow-up	266 812.3	289	1.47 (1.21-1.79)	266 433.6	222	1.45 (1.16-1.81)	265 334.2	67	1.54 (1.03-2.30)
Prevalent diabetes at enrollment	172 029.8	199	1.59 (1.28-1.97)	171 714.9	151	1.55 (1.21-1.98)	170 884.3	48	1.74 (1.12-2.69)
Incident diabetes during follow-up	94 782.5	90	1.28 (0.99-1.66)	94 718.7	71	1.30 (0.97-1.74)	94 449.8	19	1.22 (0.70-2.11)
Female participants (n =34 786)									
No diabetes	211 491.0	114	1 [Reference]	211 290.8	92	1 [Reference]	210 642.9	22	1 [Reference]
Diabetes at enrollment or follow-up	176 555.8	190	1.59 (1.24-2.04)	176 384.6	153	1.53 (1.16-2.02)	175 556.1	37	1.87 (1.05-3.30)
Prevalent diabetes at enrollment	113 689.2	127	1.65 (1.26-2.17)	113 547.0	101	1.57 (1.16-2.13)	112 936.3	26	2.06 (1.11-3.83)
Incident diabetes during follow-up	62 866.6	63	1.49 (1.09-2.05)	62 837.6	52	1.47 (1.04-2.09)	62 619.8	11	1.55 (0.73-3.28)
Male participants (n = 19 811)									
No diabetes	119 544.8	83	1 [Reference]	119 345.7	58	1 [Reference]	119 047.1	25	1 [Reference]
Diabetes at enrollment or follow-up	90 256.5	99	1.27 (0.93-1.75)	90 049.0	69	1.27 (0.87-1.86)	89 778.1	30	1.27 (0.72-2.26)
Prevalent diabetes at enrollment	58 340.6	72	1.50 (1.06-2.11)	58 167.9	50	1.49 (0.99-2.27)	57 948.1	22	1.50 (0.80-2.81)
Incident diabetes during follow-up	31 915.9	27	0.95 (0.61-1.48)	31 881.1	19	0.95 (0.56-1.62)	31 830.0	8	0.92 (0.41-2.09)

Abbreviation: HR, hazard ratio.

^a^ Participants without diabetes who did not attend follow-up 3 were censored at their age at the last follow-up where they participated.

^b^ Participants with incident diabetes contribute follow-up time to the no diabetes and diabetes during follow-up exposure groups. In total, 17 colorectal cancer cases developed cancer before diabetes (14 colon and 3 rectal cases), and consequently these cases’ person-years before cancer diagnosis are counted in the reference group for this table.

^c^ Analyses adjusted for enrollment source, race and ethnicity, sex, health insurance status, colorectal cancer screening at enrollment, smoking status, education, income, alcohol intake, body mass index, and family history of colorectal cancer.

### Effect Modification by CRC Risk Factors for the Association Between Diabetes and CRC Risk

**Figure 1.**
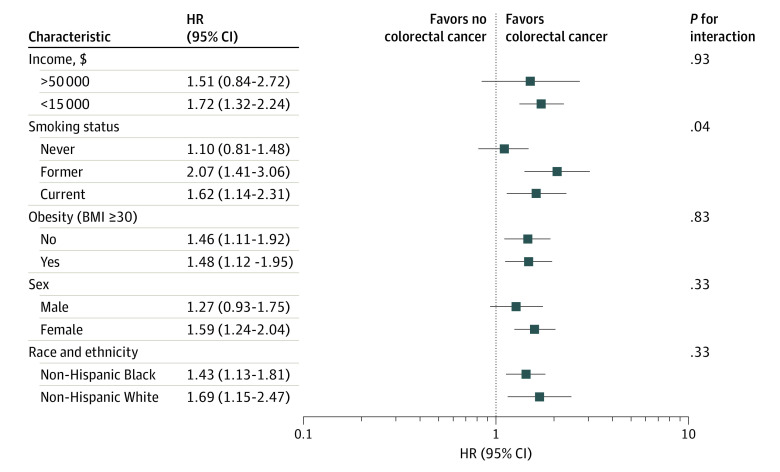
Associations Between Diabetes and Incident Colorectal Cancer (CRC), Stratified by Risk Factors for CRC^a,b^ Data presented as hazard ratios (HRs; with 95% CIs) for incident CRC in persons with diabetes compared with persons without diabetes. Diabetes includes prevalent diabetes at enrollment and incident diabetes during follow-up. BMI indicates body mass index (calculated as weight in kilograms divided by height in meters squared); CRC, colorectal cancer. ^a^Participants without diabetes who did not attend follow-up 3 were censored at their age at the last follow-up where they participated. ^b^Analyses adjusted for enrollment source, race and ethnicity, sex, health insurance status, CRC screening at enrollment, smoking status, education, income, alcohol intake, BMI (calculated as weight in kilograms divided by height in meters squared), and family history of colorectal cancer.

There was little evidence for effect modification between diabetes and CRC risk by race and ethnicity, sex, obesity, or income (race and ethnicity *P* for interaction = .33; sex *P* for interaction = .33; obesity *P* for interaction = .83; income *P* for interaction = .93) (Figure 1; eTable 4 in Supplement 1). Associations between diabetes and CRC risk varied by smoking status where diabetes was associated with increased risk among former and current smokers but not for never smokers (*P* for interaction = .04), potentially due to higher CRC screening participation among never smokers (Table 1).

### Differences in the Association Between Diabetes Status and CRC Risk by Participation in Screening

**Figure 2.**
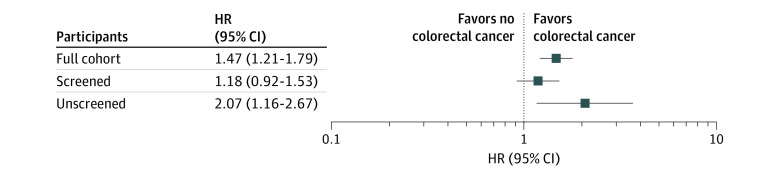
Association Between Diabetes and Risk for Colorectal Cancer (CRC) Stratified by Colonoscopy Screening Status Data presented as hazard ratios (HRs; with 95% CIs) for incident CRC in persons with diabetes compared with persons without diabetes. Analyses adjusted for enrollment source, race and ethnicity, sex, health insurance status, CRC screening at enrollment, smoking status, education, income, alcohol intake, body mass index, and family history of colorectal cancer. HR indicates hazard ratio.

The association between diabetes status and CRC risk differed by participation in colonoscopy screening (Figure 2, eTable 5 in Supplement 1). Among participants who had never undergone colonoscopy, we see the greatest association between diabetes and CRC risk with an HR of 2.07 (95% CI, 1.16-3.67). Conversely, among participants who reported screening by colonoscopy, the HR for the association between diabetes and CRC was reduced to 1.18 (95% CI, 0.92-1.53).

### CRC Risk by Duration of Diabetes

We evaluated whether the association between diabetes and CRC varied by diabetes duration (Table 3). Compared with diabetes duration of 5 to 10 years, participants with duration of 2 to 5 years were at increased CRC risk (HR, 2.55; 95%, 1.77-3.67). Decreased CRC risk was detected in those with diabetes duration 10 years or longer. Associations between diabetes duration were similar for colon and rectal cancer.

**Table 3.** Associations Between Diabetes Duration and Colorectal Cancer Incidence, Among Participants With Diabetes Diagnoses^a^

All diabetes diagnosis	Diabetes duration, median (IQR), y	Cohort, No.	Cases, No.	HR (95% CI)^b^
Total patients				
≥2 to <5	3.9 (3.2-4.5)	1662	48	2.55 (1.77-3.67)
≥5 to <10	7.8 (6.5-8.9)	5969	73	1 [Reference]
≥10 to <15	12.3 (11.1-13.6)	7376	60	0.60 (0.42-0.84)
≥15	20.3 (17.3-25.6)	10 464	84	0.48 (0.35-0.66)
Prevalent diabetes diagnosis at enrollment				
≥2 to <5	4.1 (3.4-4.6)	100	9	3.49 (1.69-7.20)
≥5 to <10	8.3 (6.8-9.3)	817	46	1 [Reference]
≥10 to <15	13.2 (11.8-14.1)	3473	56	0.16 (0.11-0.23)
≥15	20.5 (17.4-25.8)	10 268	84	0.05 (0.04-0.08)
Incident diabetes diagnosis during study follow-up				
≥2 to <5	3.9 (3.2-4.5)	1562	39	5.30 (3.24-8.67)
≥5 to <10	7.8 (6.5-8.8)	5152	27	1 [Reference]
≥10	11.7 (10.8-13.0)	4099	4	0.15 (0.05-0.43)

Abbreviation: HR, hazard ratio.

^a^ Analysis includes participants who reported a diabetes diagnosis and had 2 or more years of follow-up after diabetes diagnosis.

^b^ Analyses adjusted for enrollment source, race and ethnicity, sex, health insurance status, CRC screening at enrollment, smoking status, education, income, alcohol intake, body mass index, and family history of colorectal cancer.

## Discussion

In a cohort composed primarily of participants who self-report African American identity and low-income, diabetes diagnosis is consistently associated with increased risk of CRC. Associations are greatest for the subgroups of participants with recent diabetes diagnosis and those without recent colonoscopy, highlighting the importance of CRC screening as a potential disruptor of the adverse outcomes of metabolic dysregulation to increase CRC.

The association between diabetes and elevated risk for CRC may be explained by biological mechanisms including hyperglycemia and hyperinsulinemia. CRC tumor cells favor glycolytic metabolism,^19^ and hence hyperglycemia may support tumorigenesis via provision of additional glucose required to support cell proliferation.^8^ Likewise, hyperinsulinemia may increase CRC risk by facilitating glucose uptake in tumor cells and by interacting with insulin receptors to activate proliferative signaling pathways.^13^ A recent meta-analysis found elevated CRC risk for individuals with type 2 diabetes taking exogenous insulin (relative risk, 1.69; 95% CI, 1.25, 2.27).^20^ Notably, hyperinsulinemia occurs early in diabetes progression (including prediabetes) and does not persist indefinitely, as endogenous insulin production eventually declines due to pancreatic exhaustion. The hypothesis that hyperinsulinemia in early diabetes increases CRC risk is supported by the increased CRC risk observed in the current study among participants with shorter duration of diabetes. Overall, those with diabetes for 2 to 5 years had positive associations with CRC risk compared with those with diabetes from 5 to 10 years. Several previous studies also report an increased CRC risk associated with shorter diabetes duration,^21,22^ although other studies report longer duration of diabetes is associated with higher CRC risk.^23,24,25,26,27^

Evidence from the present study also suggests access to health care may change the association between diabetes diagnosis and CRC. Our finding that shorter duration of diabetes is associated with elevated CRC risk may be partially attributable to increased interactions with the health care system after diabetes diagnosis, allowing for cancer screening opportunities. The increase in CRC testing may initially increase CRC through the detection of prevalent CRCs and over the long term may result in reduced risk of CRC due to screening’s preventive function against CRC.^28^ Additional support for this hypothesis is that the association between diabetes and CRC risk is most apparent among those without participation in CRC screenings. Associations in the nonscreened sample are more likely to measure the natural history of the metabolic influence of diabetes on CRC.

Additionally, associations between diabetes and CRC risk are greatest in former and current smokers and null among never smokers. CRC screening rates are higher among never smokers, providing further evidence of the importance of preventative screening and health behaviors to reduce the metabolic influence of diabetes on CRC. Moreover, cigarette smoking is inflammatory^29^ and may exacerbate the inflammatory mechanisms of diabetes hypothesized to contribute to CRC. Although 1 study^30^ similarly found that smoking strengthens the association between diabetes and CRC, another found the opposite.^31^ A third study^32^ identified no interaction. The large number of current smokers in the SCCS relative to other cohorts may have led to greater statistical power to identify interactions with diabetes.

We did not observe effect modification by BMI for the association between diabetes and CRC risk. Consistent with our finding, previous literature shows limited evidence of modification by BMI.^27,32,33^ Associations were consistent by sex, race and ethnicity, income, and cancer site, although CIs are wider for rectal cancer associations, possibly reflecting small case numbers. Moderately lesser associations between diabetes and rectal cancer have been reported previously.^31,34,35^

### Limitations and Strengths

This study had limitations. In our analysis, there were considerable missing data for our primary exposure of diabetes diagnosis. To mitigate the effect of unreported diabetes on effect estimates, we excluded from regression models participants without diabetes at enrollment who did not participate in any follow-up, and censored individuals without diabetes who did not participate in the most recent study follow-up. Importantly, unreported diabetes may bias associations with incident CRC toward the null. In support of this, an analysis including participants with missing follow-up demonstrated lesser associations, although in the same direction and with largely overlapping CIs compared with the analysis excluding these participants. In addition to missing data, undiagnosed diabetes within the study population is a significant issue in diabetes research^36^ and may attenuate associations toward the null. Our study relies on self-reported diabetes diagnosis, as did many previous studies,^31,37^ instead of medical records^35^ or blood testing. The SCCS has previously reported low sensitivity of self-report diabetes.^18^ Additionally, the study sample included a majority of participants of low SES, a population associated with higher rates of undiagnosed diabetes.^38^ Differential misclassification of diabetes by race and ethnicity is an additional concern. In the SCCS, a study of a random sample of 789 participants found that 4% of White participants and 10% to 20% of Black participants had elevated HbA_1c_ despite reporting negative diabetes status, suggesting differential rates of undetected diabetes.^18,39^ These limitations suggest some of the reported associations may be biased toward the null and racial and SES differences may be obscured. Another potential limitation is that, similar to other publications,^31,37^ we were unable to fully differentiate between type 1 and type 2 diabetes. However, we excluded diabetes diagnoses which occurred before age 30 to remove likely type 1 diabetes. Furthermore, for participants who underwent colonoscopy, we were unable to determine the reason for this procedure (eg, screening, diagnosis, or surveillance), which may influence the association with CRC risk. Additionally, there is the possibility of type I error in our analysis due to multiple comparisons.

This study has several strengths including the prospective study design and linkage to state cancer registries and the National Death Index to facilitate near complete capture of incident CRC. The SCCS represents a population that, despite high rates of diabetes and CRC, has been underrepresented in the literature.

## Conclusions

These findings contribute evidence of an association between diabetes and CRC risk. These associations were greatest for those with recent diabetes and those who did not participate in colonoscopy. Increased interactions with the health care system following a diabetes diagnosis, including increased referrals to CRC screening, may be important for mitigating the harm of diabetes-related metabolic dysfunction, particularly in early diabetes, on CRC risk.

## Notes

Supplement 1. **eFigure. **Schematic Representing the Follow-Up Data Collection of the Southern Community Cohort Study **eTable 1. **Characteristics of Southern Community Cohort Study Participants With and Without Incident Colorectal Cancer **eTable 2. **Characteristics of Southern Community Cohort Study Participants Stratified by Timing of Diabetes Diagnosis Among the Full Analytic Sample **eTable 3. **Associations Between Diabetes and Colorectal Cancer Incidence Among the Full Analytic Sample, Stratified by Anatomic Site **eTable 4. **Associations Between Diabetes and Colorectal Cancer Incidence Stratified by Risk Factors for Colorectal Cancer **eTable 5. **Associations Between Diabetes and Colorectal Cancer Incidence, Stratified by Colonoscopy Use

Supplement 2.
Data Sharing Statement
