# Supplementary material for: Type 2 Diabetes and Colorectal Cancer Risk
Source: JAMA Netw Open. 2023 Nov 14;6(11):e2343333. doi: 10.1001/jamanetworkopen.2023.43333 (PMC10646729; doi:10.1001/jamanetworkopen.2023.43333)
Supplement: Supplement 1. — eFigure. Schematic Representing the Follow-Up Data Collection of the Southern Community Cohort Study eTable 1. Characteristics of Southern Community Cohort Study Participants With and Without Incident Colorectal Cancer eTable 2. Characteristics of Southern Community Cohort Study Participants Stratified by Timing of Diabetes Diagnosis Among the Full Analytic Sample eTable 3. Associations Between Diabetes and Colorectal Cancer Incidence Among the Full Analytic Sample, Stratified by Anatomic Site eTable 4. Associations Between Diabetes and Colorectal Cancer Incidence Stratified by Risk Factors for Colorectal Cancer eTable 5. Associations Between Diabetes and Colorectal Cancer Incidence, Stratified by Colonoscopy Use [file jamanetwopen-e2343333-s001.pdf]

Supplemental Online Content

Lawler T, Walts ZL Steinwandel M, et al. Type 2 diabetes and colorectal cancer risk. *JAMA Netw Open*. 2023;6(11):e2341533. doi:10.1001/jamanetworkopen.2023.41533

eFigure. Schematic Representing the Follow-Up Data Collection of the Southern Community Cohort Study
eTable 1. Characteristics of Southern Community Cohort Study Participants With and Without Incident Colorectal Cancer
eTable 2. Characteristics of Southern Community Cohort Study Participants Stratified by Timing of Diabetes Diagnosis Among the Full Analytic Sample
eTable 3. Associations Between Diabetes and Colorectal Cancer Incidence Among the Full Analytic Sample, Stratified by Anatomic Site
eTable 4. Associations Between Diabetes and Colorectal Cancer Incidence Stratified by Risk Factors for Colorectal Cancer
eTable 5. Associations Between Diabetes and Colorectal Cancer Incidence, Stratified by Colonoscopy Use

This supplemental material has been provided by the authors to give readers additional information about their work.

© 2023 Lawler T et al. *JAMA Network Open.*

eFigure. Schematic Representing the Follow-Up Data Collection of the Southern Community Cohort Study

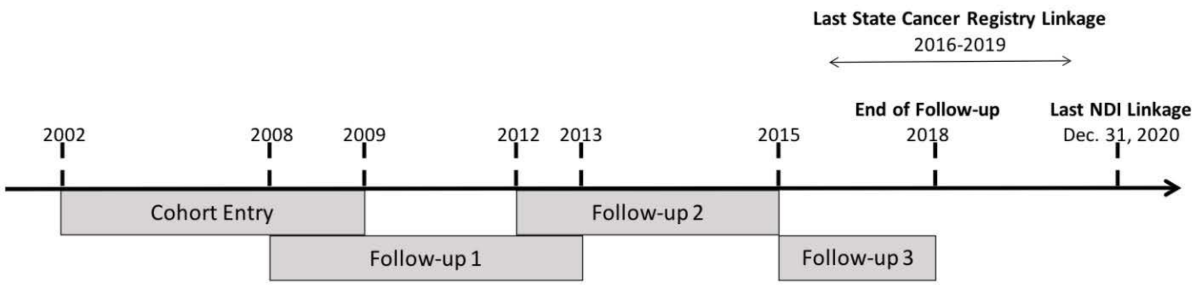

This schematic represents the timing of cohort entry and each of the three follow-up surveys. Incident diagnosis of colorectal cancer is captured through linkages with state cancer registries and the National Death Index. The most recent linkages with state cancer registries occurred in 2016-2019, depending on the state. The most recent linkages with the National Death Index was through December 31, 2020.

Abbreviations: NDI – National Death Index

© 2023 Lawler T et al. *JAMA Network Open.*

eTable 1. Characteristics of Southern Community Cohort Study Participants With and Without Incident Colorectal Cancer (CRC) (n = 54,597) [a]				
	**Cohort (n = 54,597)**		**No CRC (n = 54,111)**	**Incident CRC (n = 486)**
Enrollment age, median (25th-75th percentile), years	51 (46-58)		51 (46-58)	55 (49-62)
Male sex	19,811 (36%)		19,629 (36%)	182 (37%)
Enrollment Source				
Community Health Center	45,431 (83%)		45,033 (83%)	398 (82%)
General Population	9,166 (17%)		9,078 (17%)	88 (18%)
Race				
African American	36,170 (66%)		35,835 (66%)	335 (69%)
White	16,277 (30%)		16,143 (30%)	134 (28%)
Other [b]	2,150 (4%)		2,133 (4%)	17 (3%)
Educational attainment				
< High school	14,353 (26%)		14,209 (26%)	144 (30%)
High school	17,664 (32%)		17,503 (32%)	161 (33%)
> High school	22,580 (41%)		22,399 (41%)	181 (37%)
Household income, $				
< 15,000	28,792 (53%)		28,513 (53%)	279 (57%)
15,000-49,999	19,735 (36%)		19,583 (36%)	152 (31%)
≥ 50,000	6,070 (11%)		6,015 (11%)	55 (11%)
Insurance, yes	34,747 (64%)		34,445 (64%)	302 (62%)
Colorectal cancer screening, yes	18,229 (33%)		18,098 (33%)	131 (27%)
Family history of colorectal cancer	3,712 (7%)		3,673 (7%)	39 (8%)
Obese body mass index, ≥ 30 kg/m^2	26,616 (49%)		26,362 (49%)	254 (52%)
Smoking status				
Never	21,630 (40%)		21,428 (40%)	202 (42%)
Former	13,457 (25%)		13,317 (25%)	140 (29%)
Current	19,510 (36%)		19,366 (36%)	144 (30%)
Non and moderate alcohol consumers [c]	46,443 (85%)		46,023 (85%)	420 (86%)
Physical activity, median (25th-75th percentile), MET-hrs/day	17 (9-30)		17 (9-29)	17 (9-28)
Sedentary time, median (25th-75th percentile), hours	9 (6-12)		9 (6-12)	8 (6-12)

[a] Data presented as N (%) for categorical variables, and median (IQR) for continuous variables

[b] Includes Hispanic/Latino, Asian or Pacific Islander, American Indian or Alaska Native, participants of more than one race, and participants from other racial or ethnic groups

[c] Non to moderate alcohol consumption defined as alcohol intake ≤1 drink/day for women or ≤2 drink/day for men.

Abbreviations: CRC – colorectal cancer; MET – metabolic equivalent

© 2023 Lawler T et al. *JAMA Network Open.*

eTable 2. Characteristics of Southern Community Cohort Study Participants Stratified by Timing of Diabetes Diagnosis Among the Full Analytic Sample (n = 73,477)[a]

		Diabetes (n = 25,992)	
	**No diabetes (n = 47,485)**	**Prevalent (n = 14,998)**	**Incident (n = 10,994)**
Enrollment age, median (25th-75th percentile), years	49 (44-56)	54 (48-61)	50 (45-56)
Male sex	21,272 (45%)	5,342 (36%)	3,731 (34%)
Enrollment Source			
Community Health Center	41,183 (87%)	13,247 (88%)	9,275 (84%)
General Population	6,302 (13%)	1,751 (12%)	1,719 (16%)
Race			
African American	31,162 (66%)	10,578 (71%)	8,335 (76%)
White	14,420 (30%)	3,818 (25%)	2,318 (21%)
Other [b]	1,903 (4%)	602 (4%)	341 (3%)
Educational attainment			
< High school	12,777 (27%)	5,059 (34%)	3,066 (28%)
High school	15,988 (34%)	4,827 (32%)	3,721 (34%)
> High school	18,720 (39%)	5,112 (34%)	4,207 (38%)
Household income, $			
< 15,000	25,858 (54%)	9,071 (60%)	6,013 (55%)
15,000-49,999	16,706 (35%)	4,977 (33%)	4,096 (37%)
≥ 50,000	4,921 (10%)	950 (6%)	885 (8%)
Insurance, yes	27,164 (57%)	10,084 (67%)	6,644 (60%)
Colorectal cancer screening, yes	12,881 (27%)	5,731 (38%)	3,363 (31%)
Family history of colorectal cancer	2,991 (6%)	1,063 (7%)	689 (6%)
Obese body mass index, ≥ 30 kg/m^2	16,740 (35%)	9,874 (66%)	6,116 (56%)
Smoking status			
Never	16,228 (34%)	6,234 (42%)	4,227 (38%)
Former	9,361 (20%)	4,398 (29%)	2,474 (23%)
Current	21,896 (46%)	4,366 (29%)	4,293 (39%)
Non and moderate alcohol consumers [c]	37,413 (79%)	13,732 (92%)	9,166 (83%)
Physical activity, median (25th-75th percentile), MET-hrs/day	19 (10-33)	15 (8-25)	18 (10-31)
Sedentary time, median (25th-75th percentile), hours	8 (6-12)	9 (6-12)	9 (6-12)
Age at CRC diagnosis, median (25th-75th percentile), years (n = 785)	60 (56-67)	65 (58-71)	61 (54-70)
CRC site (n = 785)			
Colon	378 (76%)	151 (76%)	71 (79%)
Rectum	118 (24%)	48 (24%)	19 (21%)
Tumor stage (n = 565)			
0	15 (4%)	7 (4%)	2 (3%)
I	86 (25%)	44 (28%)	19 (30%)
II	81 (24%)	36 (23%)	14 (22%)
III	70 (20%)	39 (25%)	15 (23%)
IV	90 (26%)	33 (21%)	14 (22%)

[a] Data presented as N (%) for categorical variables, and median (25th-75th percentile) for continuous variables. The table includes participants without diabetes who were missing data from follow-ups 1-3 (n = 18,880).

[b] Includes Hispanic/Latino, Asian or Pacific Islander, American Indian or Alaska Native, participants of more than one race, and participants from other racial or ethnic groups

[c] Non to moderate alcohol consumption defined as alcohol intake ≤1 drink/day for women or ≤2 drink/day for men.
Abbreviations: CRC – colorectal cancer; MET – metabolic equivalent

© 2023 Lawler T et al. *JAMA Network Open.*

eTable 3. Associations Between Diabetes and Colorectal Cancer Incidence Among the Full Analytic Sample, Stratified by Anatomic Site (n = 73,477) [a]									
	**Colorectal cancer incidence**			**Colon cancer incidence**			**Rectal cancer incidence**		
	**Person-years**	**Cases [b]**	**HR [c] (95% CI)**	**Person-years**	**Cases [b]**	**HR [c] (95% CI)**	**Person-years**	**Cases [b]**	**HR [c] (95% CI)**
**All participants (n = 73,477)**									
No diabetes (ref)	629,145.2	496	1	628,199.0	378	1	625,907.3	118	1
Diabetes at baseline or follow-up [d]	266,812.3	289	1.14 (0.98-1.33)	266,433.6	222	1.12 (0.94-1.33)	265,334.2	67	1.21 (0.88-1.66)
Diabetes at baseline [d]	172,029.8	199	1.22 (1.03-1.46)	171,714.9	151	1.19 (0.97-1.45)	170,884.3	48	1.36 (0.95-1.94)
Diabetes during follow-up [d]	94,782.5	90	1.00 (0.79-1.25)	94,718.7	71	1.01 (0.78-1.30)	94,449.8	19	0.96 (0.58-1.57)
**Females (n = 43,132)**									
No diabetes (ref)	359,046.2	253	1	358,575.3	198	1	357,300.1	55	1
Diabetes at baseline or follow-up [d]	176,555.8	190	1.25 (1.03-1.53)	176,384.6	153	1.27 (1.02-1.59)	175,556.1	37	1.19 (0.77-1.84)
Diabetes at baseline [d]	113,689.2	127	1.31 (1.04-1.64)	113,547.0	101	1.31 (1.02-1.69)	112,936.3	26	1.30 (0.79-2.13)
Diabetes during follow-up [d]	62,866.6	63	1.17 (0.88-1.55)	62,837.6	52	1.21 (0.89-1.65)	62,619.8	11	0.99 (0.52-1.92)
**Males (n = 30,345)**									
No diabetes (ref)	270,099.0	243	1	269,623.7	180	1	268,607.3	63	1
Diabetes at baseline or follow-up [d]	90,256.5	99	0.96 (0.75-1.24)	90,049.0	69	0.88 (0.66-1.18)	89,778.1	30	1.24 (0.78-1.97)
Diabetes at baseline [d]	58,340.6	72	1.11 (0.84-1.47)	58,167.9	50	1.01 (0.72-1.40)	57,948.1	22	1.45 (0.86-2.44)
Diabetes during follow-up [d]	31,915.9	27	0.73 (0.49-1.09)	3,1881.1	19	0.67 (0.42-1.08)	31,830.0	8	0.90 (0.43-1.91)

[a] Analyses include participants without diabetes who were missing data from follow-ups 1-3 (n = 18,880). Censoring of participants without diabetes who were missing data from follow-up 3 (n = 13,131) is not performed.

[b] Cases with incident diabetes contribute follow-up time to the 'no-diabetes' and 'diabetes during follow-up' exposure groups.

[c] Analyses adjusted for enrollment source, race, sex, health insurance status, CRC screening at enrollment, smoking status, education, income, alcohol intake, body mass index, and family history of colorectal cancer.

[d] In total, 17 colorectal cancer cases developed cancer before diabetes (14 colon and 3 rectal cases), and consequently these cases' person-years before cancer diagnosis are counted in the reference group for this table.

Abbreviations: CI – confidence interval; CRC – colorectal cancer; HR – hazard ratio; ref - reference.

© 2023 Lawler T et al. *JAMA Network Open.*

**eTable 4. Associations Between Diabetes and Colorectal Cancer Incidence Stratified by Risk Factors for Colorectal Cancer (n = 54,597) [a]**			
	**Person-years**	**Cases [b]**	**HR [c] (95%CI)**
**Race**			
African American	595,752.2	335	1.43 (1.13-1.81)
Non-Hispanic White	236,738.8	134	1.69 (1.15-2.47)
P-interaction			*.33*
**Sex**			
Male	300,057.8	182	1.27 (0.93-1.75)
Female	564,602.5	304	1.59 (1.24-2.04)
P-interaction			*.33*
**Obesity (≥ 30kg/m^2)**			
No	397,524.8	232	1.46 (1.11-1.92)
Yes	467,135.5	254	1.48 (1.12-1.95)
P-interaction			*.83*
**Smoking status**			
Never	354,847.5	202	1.10 (0.81-1.48)
Former	221,198.1	140	2.07 (1.41-3.06)
Current	288,614.7	144	1.62 (1.14-2.31)
P-interaction			*.04*
**Household income (per year)**			
< $15,000	460,276.6	279	1.72 (1.32-2.24)
≥ $50,000	90,217.1	55	1.51 (0.84-2.72)
P-interaction			*.93*

[a] Participants without diabetes who did not attend follow-up 3 were censored at their age at the last follow-up where they participated.

[b] Cases with incident diabetes contribute follow-up time to the 'no-diabetes' and 'diabetes' exposure groups.

[c] Analyses adjusted for enrollment source, race, sex, health insurance status, CRC screening at enrollment, smoking status, education, income, alcohol intake, body mass index, and family history of colorectal cancer.

Abbreviations: HR – hazard ratio; CI – confidence interval

© 2023 Lawler T et al. *JAMA Network Open.*

eTable 5. Associations Between Diabetes and Colorectal Cancer Incidence, Stratified by Colonoscopy Use[a]						
	**Participants who did not participate in colonoscopy during cohort follow-up 2 or 3 [b]**			**Participants screened for diabetes and by colonoscopy during cohort follow-up 2 or 3 [c]**		
	**Person-years**	**Cases [d]**	**HR [e] (95% CI)**	**Person-years**	**Cases [d]**	**HR [e] (95% CI)**
No diabetes (ref)	77,810.3	24	1	198,843.5	133	1
Diabetes at enrollment or follow-up	38,328.3	31	2.07 (1.16-3.67)	141,116.5	141	1.18 (0.92-1.53)
Prevalent diabetes at enrollment	20,292.3	19	2.37 (1.22-4.60)	78,027.2	80	1.24 (0.93-1.67)
Incident diabetes during follow-up	18,036.0	12	1.77 (0.86-3.61)	63,089.4	61	1.12 (0.82-1.53)

[a] Participants without diabetes who did not attend follow-up 3 were censored at their age at the last follow-up where they participated.

[b] This analysis includes participants who completed cohort follow-up 2 or 3 interviews and reported never undergoing colonoscopy at their most recent study interview.

[c] This analysis includes participants who completed cohort follow-up 2 or 3 interviews and reported undergoing colonoscopy. Participants considered screened for diabetes were those who self-reported a diabetes diagnosis or who self-reported ever having been screened by a physician for diabetes.

[d] Participants with incident diabetes contribute follow-up time to the ‘no-diabetes’ and ‘diabetes during follow-up’ exposure groups.

[e] Analyses adjusted for enrollment source, race, sex, health insurance status, CRC screening at enrollment, smoking status, education, income, alcohol intake, body mass index, and family history of colorectal cancer.

Abbreviations: CI – confidence interval; CRC – colorectal cancer; HR – hazard ratio; ref – reference.

© 2023 Lawler T et al. *JAMA Network Open.*
