# Supplementary material for: Type 2 Diabetes and Colorectal Cancer Risk
Source: JAMA Netw Open. 2023 Nov 14;6(11):e2343333. doi: 10.1001/jamanetworkopen.2023.43333 (PMC10646729; doi:10.1001/jamanetworkopen.2023.43333)
Supplement: Supplement 2. — Data Sharing Statement [file jamanetwopen-e2343333-s002.pdf]

Data Sharing Statement

Lawler. Type 2 Diabetes and Colorectal Cancer Risk. *JAMA Netw Open*. Published November 15, 2023. doi:10.1001/jamanetworkopen.2023.43333

Data
Data available: No

Additional Information
Explanation for why data not available: Data is available to qualified investigators by submitting a concept proposal to the Southern Community Cohort Study Data & Biospecimen Review Committee. More information is available at: https://www.southerncommunitystudy.org/
